# Determination of Pyrrolizidine Alkaloids in Tea and Herbal Infusions by LC-MS/MS

**DOI:** 10.3390/foods15203598

**Published:** 2026-10-10

**Authors:** Ana Catarina Sousa, Daniela Pereira, Ana R. L. Ribeiro, Virgínia M. F. Gonçalves, Inês Pádua, Cláudia Ribeiro, Sandra Leal

**Affiliations:** 1 Associate Laboratory i4HB—Institute for Health and Bioeconomy, CESPU University, 4585-116 Gandra, Portugal; a23452@alunos.cespu.pt (A.C.S.); virginia.goncalves@cespu.pt (V.M.F.G.); maria.silva@iucs.cespu.pt (I.P.); 2 UCIBIO—Research Unit on Applied Molecular Biosciences, Toxicologic Pathology Research Laboratory, CESPU University, 4585-116 Gandra, Portugal; 3 LSRE-LCM, ALiCE, Faculty of Engineering, University of Porto, 4200-465 Porto, Portugal; dmvpereira@fe.up.pt (D.P.); ritalado@fe.up.pt (A.R.L.R.); 4 UCIBIO—Research Unit on Applied Molecular Biosciences, Translational Toxicology Research Laboratory, CESPU University, 4585-116 Gandra, Portugal; 5 UNIPRO—Oral Pathology and Rehabilitation Research Unit, CESPU University, 4585-116 Gandra, Portugal

**Keywords:** plant toxins, pyrrolizidine alkaloids, food supply, food contaminants, food safety, human health

## Abstract

Pyrrolizidine alkaloids (PAs) and *N*-oxides (PANOs) are food-safety hazards subject to regulatory limits, and their occurrence in teas and herbal infusions (THI) represents a dietary route. This study aimed to develop and validate a liquid chromatography-tandem mass spectrometry (LC-MS/MS) method for determining five PAs and their PANOs in THI. Samples were extracted using hot acidified water to enhance PAs and PANOs extraction and subjected to solid-phase extraction (SPE) for clean-up and analyte preconcentration. Chromatographic separation was performed on a Luna^®^ PFP (2) 3 µm 100 Å column using a gradient of 10 mM ammonium formate with 0.1% formic acid in water, ethanol, and methanol. The method was validated according to international guidelines, achieving detection and quantification limits of 0.41–6.60 ng/g and 1.25–20.0 ng/g. It was applied to 53 THI samples from five European countries, primarily from Portugal as a pilot monitoring study. *N*-oxides were the predominant form, with senecionine-*N*-oxide, heliotrine-*N*-oxide, and retrorsine-*N*-oxide most frequently detected in Portuguese-marketed products. The total target PA/PANO content was approximately twofold higher in herbal infusions than in teas, with chamomile showing the highest levels, followed by lemon verbena. The validated method provides a sensitive and suitable tool for preliminary assessment of PAs and PANOs in THI.

## 1. Introduction

Pyrrolizidine alkaloids (PAs) and their *N*-oxide derivatives (PANOs) are secondary metabolites produced by plants belonging mainly to the families Asteraceae (genera *Senecio* and *Jacobaea*), Boraginaceae (genera *Heliotropium*, *Echium*, *Symphytum*, and *Borago*), and Fabaceae (mainly of the genus *Crotalaria*) [1,2]. To date, over 660 individual PAs have been characterized, exhibiting wide structural diversity [2]. The structural heterogeneity of PAs largely determines their physicochemical properties and biological activities, thereby influencing both their toxicological behavior and analytical detectability in food matrices. Among the different classes of PAs found in nature, including saturated and unsaturated PAs and their corresponding PANOs, the 1,2-unsaturated PAs are considered the most toxicologically relevant because they can be metabolically activated into reactive pyrrolic intermediates responsible for hepatotoxic, genotoxic, and carcinogenic effects [3,4].

**Figure 1:**
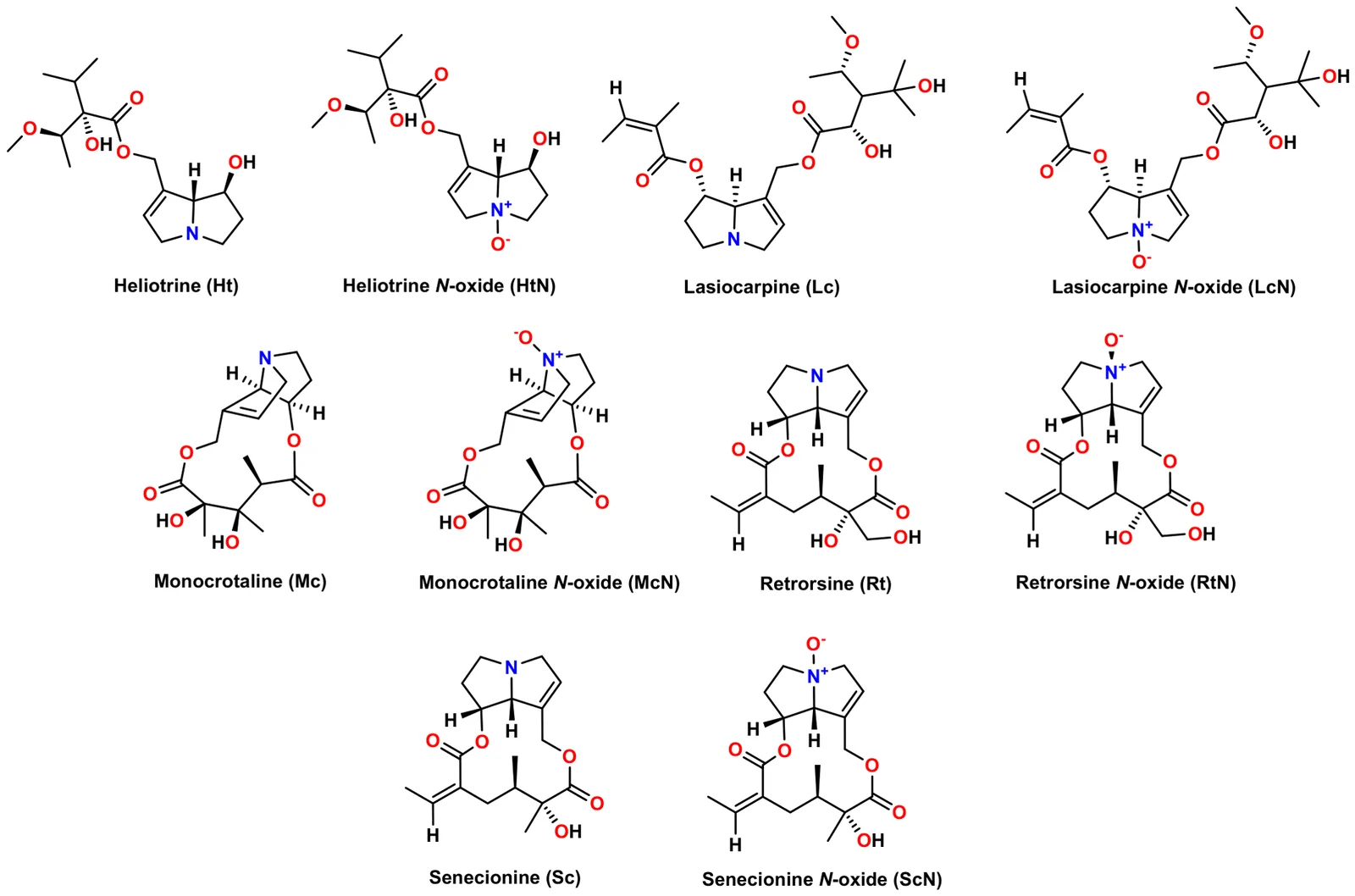
Chemical structures of the selected 1,2-unsaturated PAs and their respective PANOs. Structures were drawn using ChemDraw Professional (version 12.0).

The European Food Safety Authority (EFSA) and the German Federal Institute for Risk Assessment (BfR) have identified 1,2-unsaturated PAs and their corresponding PANOs as priority contaminants for food monitoring and risk assessment [5,6]. Based on these recommendations, the target analytes included in the present work were selected considering their reported toxicological and regulatory relevance [7]. These compounds represent the main groups of toxicologically relevant PAs, including retronecine-type PAs (e.g., senecionine, retrorsine and monocrotaline), heliotridine-type PAs (e.g., heliotrine and lasiocarpine), along with their corresponding *N*-oxide derivatives (Figure 1) [6]. Although not intrinsically toxic, PAs act as pro-toxins requiring metabolic activation, primarily by hepatic enzymes, namely cytochrome P450 monooxygenases [8]. This bioactivation generates reactive pyrrolic metabolites that irreversibly bind to DNA or proteins, promoting the accumulation of cellular adducts associated with cytotoxicity, apoptosis, and veno-occlusion disease [9], which are responsible for hepatotoxic and genotoxic effects [10], reinforcing their relevance as priority contaminants in food safety risk assessment.

The main route of human exposure to PAs is the dietary intake of contaminated food and beverages, including teas and herbal infusions (THI), as well as vegetables, honey, milk, eggs, and meat [11]. Although food of animal origin, such as milk and eggs, generally contains low concentrations, typically ranging from 0.05 to 0.17 µg/L in milk and 0.10 to 0.12 µg/kg in eggs, they may contribute to cumulative exposure, especially when consumed frequently [11]. Nevertheless, THI are considered the major source of chronic dietary exposure to PAs because of their widespread and frequent consumption [12,13]. Their popularity is linked to their content of natural bioactive compounds and the perception of health-promoting properties [14]. PAs may occur in THI either because PAs-producing plant species are intentionally used in herbal preparations or through unintentional contamination during production, including co-harvesting of PAs-producing weeds (e.g., *Senecio angustifolius*), contamination with seeds or other plant parts, cross-contamination during processing, adulteration, or botanical misidentification [15]. In addition to these endogenous contaminants, THI may also contain exogenous contaminants such as heavy metals and mycotoxins, further raising food safety concerns [16,17,18], particularly considering their frequent consumption by vulnerable population groups. As a result of the demonstrated toxicological potential of certain PAs, regulatory concern regarding human exposure through contaminated food, drinks, and herbal products has increased [19]. EFSA established a benchmark dose lower confidence limit (BMDL_10_) of 237 µg/kg body weight/day for PA-induced carcinogenic effect [19]. In comparison, the BfR recommended a more conservative maximum daily intake limit of 0.007 µg/kg body weight/day to further minimize the risks associated with chronic exposure [5], emphasizing the prevailing uncertainty and the need for robust occurrence data to support risk management decisions.

In order to mitigate the risk of human exposure and facilitate the establishment of evidence-based regulatory thresholds, several analytical methodologies have been developed for the detection and quantification of PAs and PANOs in THI [20,21,22,23,24]. A comprehensive review of the analytical methodologies available for the determination of PAs and PANOs in THI and other matrices has previously been published [24]. Since its publication, several additional analytical methods have been reported, reflecting the growing interest in monitoring these contaminants. An updated comparison of the most relevant chromatographic methods is presented in Table S1 [25,26,27,28,29,30,31,32,33,34,35,36]. Liquid chromatography coupled to tandem mass spectrometry (LC-MS/MS) remains the method of choice for PAs/PANOs due to its high sensitivity and selectivity [37,38,39,40,41,42]. However, the complexity of food and herbal matrices together with the low concentrations at which these compounds typically occur, requires efficient sample preparation procedures to minimize matrix interferences and achieve quantification. Several methods regarding analyte coverage, sample preparation procedures, validation strategies, and analytical performance have been published (Table S1). These differences highlight the need for methods that combine broad analyte determination with efficient sample preparation and accurate quantification in THI.

Therefore, the aim of this study was to develop and validate a sensitive and reliable LC-MS/MS method for the simultaneous determination of five selected 1,2-unsaturated PAs and their corresponding PANOs in THI. The validated method was subsequently applied to the analysis of commercial THI samples obtained from five European countries, with particular emphasis on the Portuguese market, where information regarding the occurrence of PAs remains scarce. This application provides preliminary data on the occurrence of PAs and their PANOs in THI.

## 2. Materials and Methods

### 2.1. Chemical Reagents and Materials

PAs, PANOs, and the internal standard (IS) were obtained from PhytoLab (Vestenbergsgreuth, Germany): Heliotrine (Ht), Heliotrine-*N*-oxide (HtN), Lasiocarpine (Lc), Lasiocarpine-*N*-oxide (LcN), Monocrotaline (Mc), Monocrotaline-*N*-oxide (McN), Retrorsine (Rt), Retrorsine-*N*-oxide (RtN), Senecionine (Sc), Senecionine-*N*-oxide (ScN) and (+)-Lycopsamine-*N*-oxide-d_7_ (LyN-d_7_).

Ethanol (EtOH, >99.8%), methanol (MeOH, >99.9%), and acetonitrile (ACN, 99.9%) HPLC gradient grade solvents were purchased from Fisher Scientific (Leicestershire, UK). Formic acid (FA) was obtained from Merck (Darmstadt, Germany). Ammonium formate was purchased from Sigma-Aldrich (>96.5%) (St. Louis, MO, USA). Ammonia 25% and sulfuric acid 95–97% were obtained from Supelco (Steinheim, Germany). Ultrapure water (UPW) was obtained from a SG Ultra Clear UV plus equipment (SG water, Barsbüttel, Germany).

Oasis^®^ MCX (Mixed-mode Cation Exchange) cartridges, 6 cc Vac Cartridges (150 mg sorbent, 30 µm particle size), were purchased from Waters^®^ (Dublin, Ireland). Glass microfiber filters with 0.70 and 1.2 μm pore sizes were purchased from VWR (Leuven, Belgium) and 0.20 μm PTFE syringe filters were obtained from Chromacol, Lab Unlimited Co. (Dublin, Ireland).

The standard stock solutions were prepared at different concentrations in EtOH and stored in amber bottles at −20 °C. A mixed standard stock solution containing all the target PAs and PANOs at a final concentration of 25 μg/mL of each analyte was prepared in UPW for method optimization.

### 2.2. Samples Collection

The selection of the samples was designed to represent the preferences of Portuguese consumers, based on a consumer preference survey [14]. Samples of THI from five European countries, primarily from Portugal were included to evaluate the applicability of the method to products with potentially different botanical origins. Although the sampling strategy focused primarily on the Portuguese market, the inclusion of these samples broadens the scope of the study and provides a preliminary assessment of potential differences in the occurrence of PAs. In addition, the THI purchased in a given country do not necessarily originate from that country but rather represent the products commercialized in local markets. In total, 53 different THI samples were collected across five European countries: Portugal (PT), France (FR), Spain (SP), Italy (IT), and Austria (AU) (Table S2). Among these, 52 were packaged in individually wrapped tea bags, and one was in loose-leaf form. From Portugal, 26 samples were collected, comprising 13 teas and 13 herbal infusions (Table S2). Eleven samples were sourced from Spain, including four teas and 7 herbal infusions. The French selection included nine tea samples, whereas four samples were obtained in Austria (one green tea and three herbal infusions) and three samples were acquired from Italian markets (two teas and one herbal infusion).

### 2.3. LC-MS/MS Analysis

After optimizing the chromatographic conditions on a LC-UV/Vis system (Shimadzu UFLC Prominence System, LC Solution (Version 1.24SP1) (Shimadzu, Tokyo, Japan), equipped with a degasser (DGU-20A5), three pumps (LC-20AD), an autosampler (SIL-20AC), a column oven (CTO-20AC), a system controller (CBM-20A), and an ultraviolet/visible (UV/Vis) detector (SPD-20A), the method was transferred to an ultra-high performance LC-MS/MS (UHPLC-MS/MS) for further analysis of PAs and PANOs in the THI samples. For that, it was used a Shimadzu UHPLC Nexera system (LC Solution Version 5.41SP1) equipped with two pumps (LC-30AD), an autosampler (SIL-30AC), a column oven (CTO-20AC), a degasser (DGU-20A5), and a system controller (CBM-20A), coupled to a triple quadrupole mass spectrometer detector (LCMS-8030, Shimadzu, Tokyo, Japan). The injection volume was 0.5 µL. Separation was carried out using a Luna^®^ PFP (2) 3 µm 100 Å column (150 × 4.60 mm) (Phenomenex, Torrance, CA, USA). The mobile phase consisted in a gradient of eluent A (10 mM aqueous ammonium formate with 0.1% FA) and eluent B (EtOH: MeOH, 30:20, *v*/*v*). The gradient elution program was as follows: 85:15 (A:B, *v*/*v*) from 0 to 6 min; a linear gradient to 55:45 (A:B, *v*/*v*) from 6 to 20 min; and maintained at 55:45 (A:B, *v*/*v*) from 20 to 30 min; and return to the initial condition (85:15; A:B, *v*/*v*) from 31 to 42 min for re-equilibration. The flow rate was maintained at 0.4 mL/min throughout the analysis. The chromatographic run was set to a total duration of 42 min, corresponding to the optimized conditions for maximizing the resolution of the target compounds, preventing co-elution of matrix components, and reducing ion suppression or enhancement effects. To optimize the MS/MS conditions, each individually prepared standard stock solution (at 1 or 10 mg/mL) was directly injected into the mass spectrometer to select the precursor ion through full scan mode and the most abundant fragments by multiple reaction monitoring (MRM). The most abundant fragment (MRM1) was used as the quantifier ion, and the second most abundant (MRM2) was used as the qualifier ion for confirmation purposes. Mass spectrometry conditions were as follows: capillary voltage, 4.5 kV; nitrogen drying gas flow, 7.5 L/min; nitrogen nebulizing gas flow, 3.0 L/min; desolvation temperature, 250 °C; and source temperature, 400 °C. The electrospray ionization (ESI) source was used in positive ion mode ([M + H]+), and argon was employed as the collision-induced dissociation (CID) gas at a pressure of 230 kPa. The dwell time was set to 100 msec for each transition. Retention times, ion transitions, and collision energies (CE) were optimized as described in Section 3.1.

### 2.4. Sample Preparation Procedure

Two grams of each THI sample were extracted by infusion in 250 mL of hot acidified distilled water (85 °C, pH 3, adjusted with sulfuric acid) for 15 min. The extract was then filtered under vacuum using a 47 mm glass microfiber filter of 1.2 µm pore size (VWR, Leuven, Belgium). Before sample cleaning and pre-concentration by solid phase extraction (SPE), a given mass of an internal standard (IS), LyN-d_7_, corresponding to a constant concentration of 4 ng/g in THI, was added to all calibration standards, quality controls, and sample extracts. The resulting filtrate was subjected to SPE using Oasis MCX cartridges using a Resprep^®^ Vacuum Manifold System with 24-port from RESTEK^®^ (Bellefonte, PA, USA). Briefly, the cartridges were conditioned with 3 mL of EtOH followed by 3 mL of UPW, after which 250 mL of the filtered extract was loaded onto the cartridge and, subsequently, washed with 4 mL of UPW. Elution was carried out using 4 mL of 5% ammonium in EtOH (*v*/*v*). Eluate (approximately 4 mL) was then filtered through a 0.22 μm PTFE syringe filter and then dried using a vacuum concentrator, CentriVap (~25 °C) with a cold trap from LABCONCO^®^ (Kansas City, MO, USA). The dried extract was reconstituted in 1 mL of EtOH, centrifuged at 4 °C, 15,000 rpm for 15 min using a Heraeus Biofuge Primo R centrifuge (Kendro Laboratory Products GmbH, Hanau, Germany), and the resulting supernatant was evaporated to dryness. Finally, the solid residue was reconstituted with 200 µL of mobile phase, consisting in 10 mM aqueous ammonium formate with 0.1% FA: EtOH: MeOH (85:9:6, *v*/*v*/*v*) and centrifuged at 4 °C, 15,000 rpm for 15 min using a Heraeus Biofuge Primo R centrifuge (Kendro Laboratory Products GmbH, Hanau, Germany). The supernatant was collected and analyzed by LC-MS/MS.

### 2.5. Method Validation

The International Council for Harmonisation (ICH) guideline [43] was used as a reference for selectivity, linearity, accuracy, intra-day and inter-day precision, limits of detection (LOD) and quantification (LOQ), and recovery. Additionally, SANTE 11312/2021 v2 guidelines and Guidance document on identification of mycotoxins and plant toxins in food and feed were also used as reference documents to support the selection and interpretation of the validation parameters [44].

Considering that there is no blank commercial reference matrix for this type of sample, for the validation, a representative validation matrix was prepared by pooling 2.00 g of dried plant material from each of 42 individual THI samples (green tea, white tea, chamomile, lemon verbena, and lemon balm), homogenized and used as a representative matrix. It should be noted that the 42 samples used for the pool were selected based on the absence of detectable levels of target PAs and PANOs in a preliminary analysis (<LOD) using LC-UV/Vis. Blank matrix and fortified (spiked) samples were compared to assess selectivity. Linearity was evaluated using matrix-match calibration curves prepared by spiking blank matrices samples in triplicate. The calibration curves were constructed using the relative peak areas calculated according to the following equation:

Relative peak area = A_A_/A_IS_

where A_A_ is the analyte peak area and the A_IS_ corresponds to the IS peak area. A total of five to seven concentration levels of analytes (1.25–80.0 ng/g), depending on the analyte, were used.

Intra-day precision was obtained from triplicate measurements of the quality controls (QC), prepared by spiking blank sample matrix at three different concentration levels (low, medium, and high). Inter-day precision was evaluated by analyzing samples at the selected concentration levels on different days. Both intra and inter-day precision was expressed as relative standard deviation, expressed as percentage (RSD %). Accuracy was assessed by calculating the percentage of agreement between the measured concentration and the corresponding nominal concentration for the QC samples. Absolute recovery was determined by comparing the analyte response obtained from the final extract of a spiked blank matrix sample, after subtraction of the response of the corresponding blank extract, with the response of a standard solution prepared at the same theoretical analyte concentration. Results were expressed as percentage (%). LOD and LOQ were initially estimated from the standard deviation of the response (σ) and the slope of the matrix-matched calibration curve (S), according to LOD = 3.3σ/S and LOQ = 10σ/S, respectively. LOQ was subsequently experimentally verified through the evaluation of accuracy and precision, with the validated LOQ concentration being included as the lowest calibration level, for which acceptable accuracy and precision were obtained. Each calibration level, including the level corresponding to the LOQ, was prepared and analyzed in triplicate. The matrix effect (ME) was evaluated by ratio of the slopes of the calibration curves obtained from matrix matched calibration curves and those prepared in solvent according to the following equation:

ME = [(*m*_matrix_/*m*_solvent_) − 1] × 100

where *m*_matrix_ corresponds to the slope of the calibration curves prepared in blank matrix, and *m*_solvent_ corresponds to the slope of the calibration curves prepared in solvent; the results are expressed as percentages (%).

## 3. Results and Discussion

### 3.1. Preliminary Assays

Before analysis by LC-MS/MS, LC-UV/Vis was initially used to optimize the method (Table S3). Although baseline resolution was achieved for all target analytes (Figure S1A), its sensitivity was insufficient for detection of the trace levels in the complex matrix (Figure S1B). Thus, the method was transferred to LC-MS/MS. Small adjustments were performed to optimize sensitivity and compatibility with mass spectrometry analysis. The MS/MS conditions were optimized, and the PAs and PANOs were measured by selecting and monitoring two transitions from those found for each parent ion. The most intense transition was used as the quantifier (MRM1), while the second most intense transition was used as the qualifier (MRM2). The criteria for accepting ion ratio deviation were established based on SANTE 11312/2021 v2 guidelines and the Guidance document on identification of mycotoxins and plant toxins in food and feed, which specify an acceptance tolerance of ±30%. However, a more conservative approach was adopted in the present study, and the ion ratio between quantifying and qualifying transitions was kept within ±20% [44,45].

**Figure 2:**
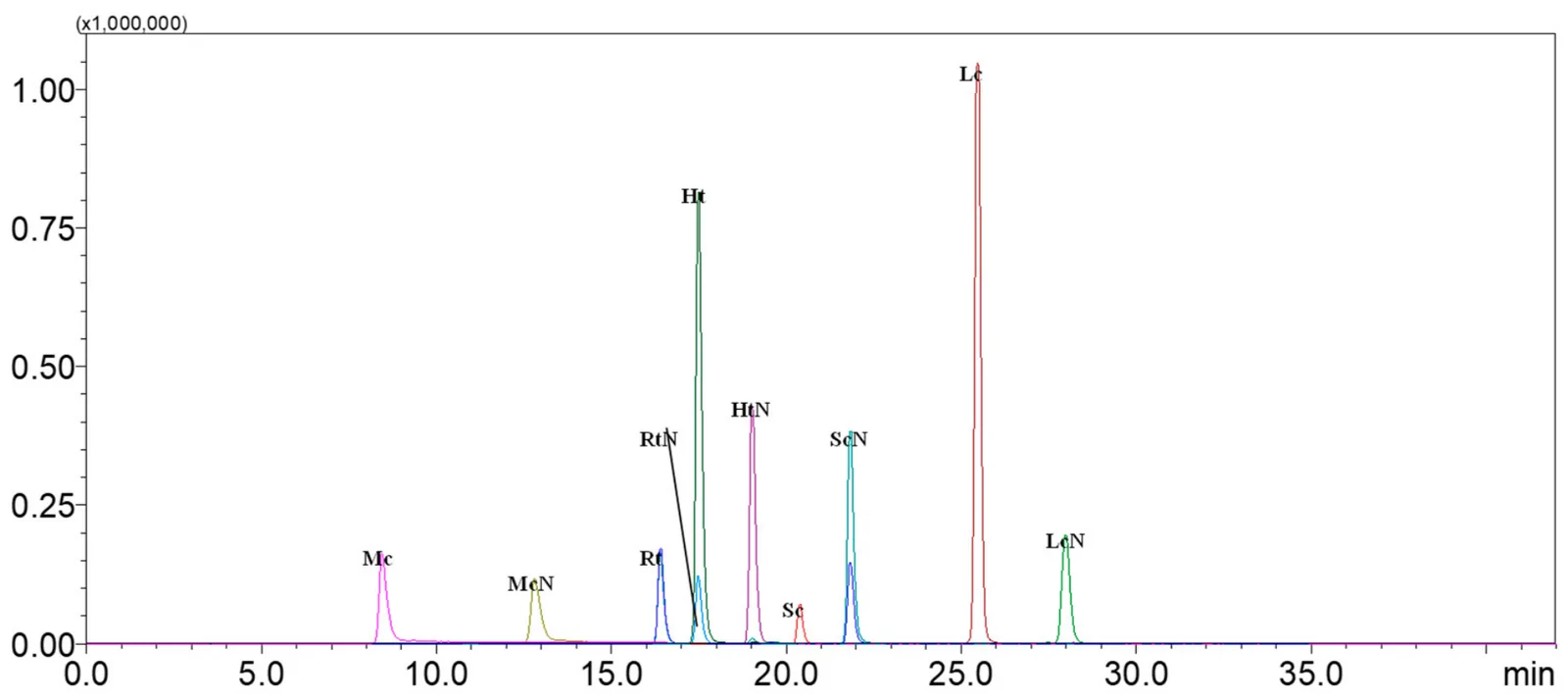
Chromatograms of an ethanolic standard solution containing 80 ng/mL of Ht: Heliotrine; HtN: Heliotrine *N*-oxide; Lc: Lasiocarpine; LcN: Lasiocarpine *N*-oxide; Mc: Monocrotaline; McN: Monocrotaline *N*-oxide; Rt: Retrorsine; RtN: Retrorsine *N*-oxide; Sc: Senecionine; ScN: Senecionine *N*-oxide. The analysis was performed using an initial mobile phase composed of 10 mM aqueous ammonium formate with 0.1% FA:EtOH:MeOH (85:9:6, *v*/*v*/*v*), in gradient elution mode, at a flow rate of 0.4 mL/min.

Following LC-MS/MS method optimization, Ht and RtN were found to coelute; however, their different precursor ions and respective quantifier and qualifier ions allowed their unequivocal identification (Table 1 and Figure 2).

### 3.2. Optimization of Sample Preparation Procedure

THI extracts were prepared using water instead of organic solvents, with extraction conditions based on consumer practices reported in a survey of THI consumers [14], in which the THI bags were predominantly immersed in boiling water. To enhance extraction efficiency, an acidification step was introduced to promote the release of PAs and PANOs from plant matrices. Although not representative of consumer practice, this step enhances the extraction of the target analytes. Comparable studies using acidified water have also reported good recoveries of the selected PAs/PANOs [31,46].

The extractions were performed using distilled water acidified with sulfuric acid to pH 3 and heated to approximately 85 °C for 15 min. Two extraction volumes (50 and 250 mL) and two sample masses were evaluated. For each volume, 0.70 g of THI (corresponding to the content of a commercial tea bag), and 2.00 g were used. After extraction, several sample preparation procedures were tested in order to maximize analyte recovery, selectivity, and sample cleanliness prior to instrumental analyses. Initially, QuEChERS-based procedures were evaluated, as recent studies have largely focused on this sample preparation technique (Table S1). Accordingly, three QuEChERS protocols were tested based on previously reported methods [47,48,49]. The parameters tested included the extraction solvent (ACN or ACN with ammonium carbonate), salt composition (anhydrous magnesium sulphate (MgSO_4_), sodium chloride (NaCl), trisodium citrate dihydrate, and disodium citrate sesquihydrate), and clean-up step, using Primary Secondary Amine (PSA) and MgSO_4_. Table 2 summarizes the main experimental parameters of the QuEChERS protocols evaluated. For all QuEChERS protocols, THI extracts were prepared from 2.00 g of sample and 50 mL of hot distilled water acidified to pH 3 with sulfuric acid (85 °C). The first QuEChERS protocol (1) followed an original QuEChERS protocol with extraction using a water-ACN solution and the addition of salts (MgSO_4_, NaCl, trisodium citrate dihydrate and disodium citrate sesquihydrate) to promote phase separation. The second QuEChERS protocol (2) comprised alkaline extraction conditions; ACN and ammonium carbonate (pH 8.5), and cleaning with PSA sorbent. The third tested QuEChERS protocol (3) used a miniaturized version of the QuEChERS approach, with smaller sample sizes and solvent volumes, but the same salt compositions and a secondary clean-up step with MgSO_4_ and PSA.

**Figure 3:**
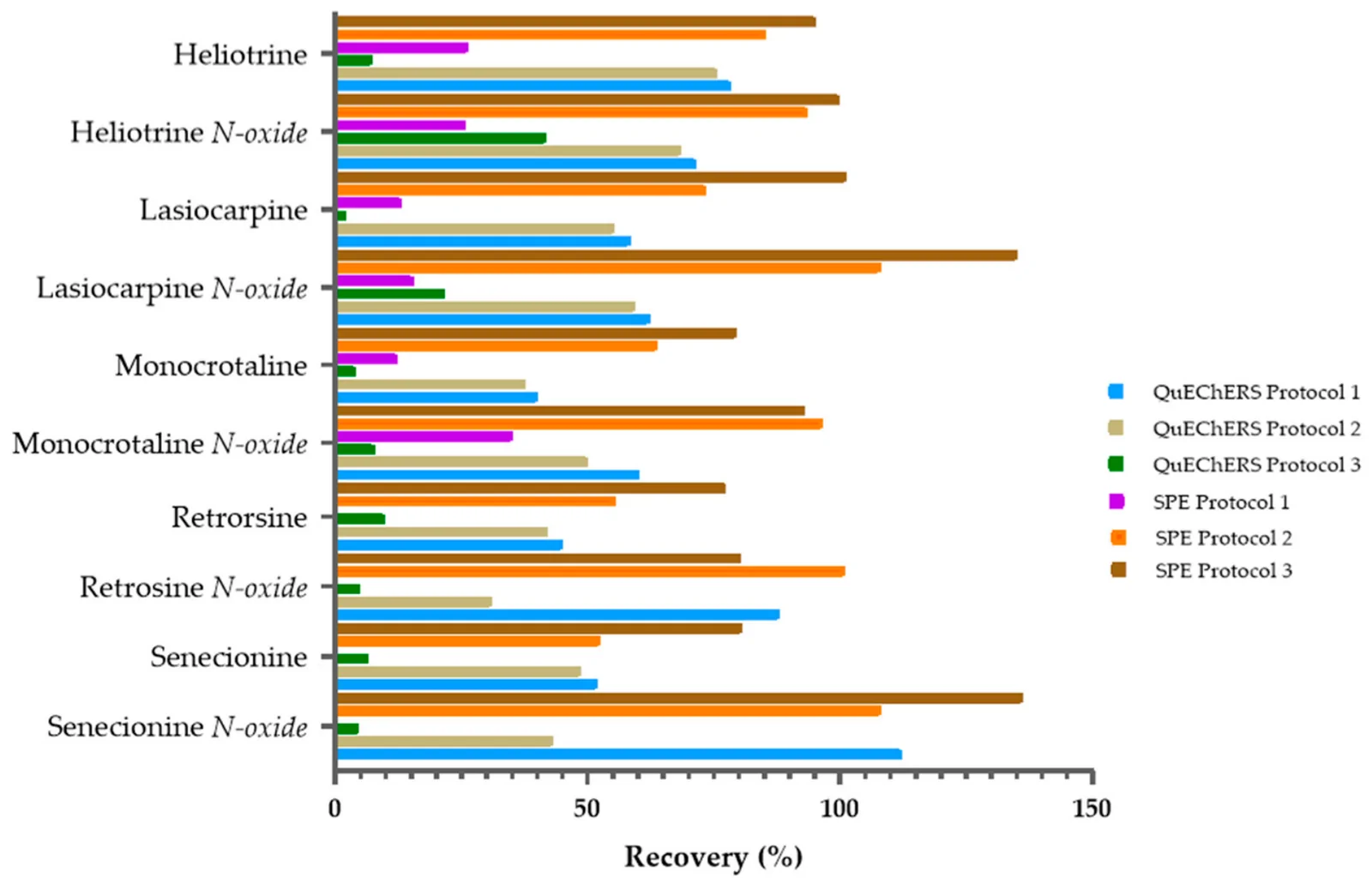
Recovery (%) of PAs and their *N*-oxides after different sample preparation procedures: solid phase extraction (SPE) and the Quick, Easy, Cheap, Effective, Rugged, Safe (QuEChERS). [SPE Protocol 1: 2.0 g samples in 50 mL (50% acidified distilled water and 50% EtOH); SPE Protocol 2: 0.7 g samples in 250 mL acidified distilled water; SPE Protocol 3: 2.0 g in 250 mL acidified distilled water)]. The graph was created using GraphPad Prism (version 8.0.2.263).

Despite the recognized simplicity and efficiency of the QuEChERS procedure, LC-MS/MS analysis revealed a poor absolute recovery (Figure 3), suggesting that the preconcentration capacity may be inadequate for quantification at trace levels, which was confirmed by the analysis of non-spiked blank samples in which no target compounds were detected when this approach was applied. This result is likely related to the very low concentrations expected in real samples, suggesting that current protocols lack sufficient preconcentration capacity to achieve the detection limits required for our analytical objectives. To overcome this limitation, a SPE procedure was tested as an alternative sample preparation procedure to improve sensitivity.

Previous studies reported the use of Mixed-mode Cation Exchange (MCX) cartridges for the extraction and concentration of PAs and PANOs from THI (Table S1) [25,31]. In the present study, MCX cartridges were selected due to their high selectivity for basic analytes and their capacity to efficiently remove acidic and polar matrix components. The SPE procedure was optimized sequentially. First, the effect of sample mass was evaluated by comparing 0.70 and 2.00 g while maintaining the remaining extraction conditions unchanged. As shown in Figure 3, the use of 2.00 g resulted in higher absolute recoveries than 0.70 g. Considering that PAs and PANOs are generally present at trace concentrations in THI, the larger sample mass increased the number of analytes extracted, thereby improving method sensitivity without compromising the clean-up capacity of the MCX cartridges. Consequently, 2.00 g was selected for the subsequent optimization experiments.

Subsequently, an extraction volume of 250 mL was selected as the optimal condition (Figure 3), as it provided higher recoveries while closely reflecting the volume typically used to prepare a cup of tea or herbal infusion, thus representing realistic consumption conditions. The larger extraction volume also improved extraction efficiency and consequently improving the overall sensitivity of the method.

Finally, the effect of extraction solvent composition was evaluated by replacing the acidified aqueous extraction solution with an acidified water–ethanol mixture. This approach led to a lower absolute recovery rate (Figure 3). This reduction is likely associated with the combined effect of the lower extraction efficiency resulting from the reduced solvent volume and possible analyte losses during sample loading onto the MCX cartridge in the presence of ethanol.

Overall, the selected extraction conditions (2.00 g sample, 250 mL acidified aqueous extraction and MCX clean-up) provided the best compromise between extraction efficiency, sample clean-up and analytical sensitivity for the determination of PAs and PANOs in THI.

### 3.3. Method Validation

Given the difficulty of obtaining a matrix completely free of the target compounds and to avoid restricting the validation to a single type of THI, a pooled representative sample matrix was prepared by combining 2.00 g of each sample in which no PAs and PANOs had been detected in a preliminary analysis (<LOD). This mixture was used as the blank matrix for validation, as no interfering peaks with the same retention time and mass-to-charge ratio as those of the analytes or the IS were detected in the preliminary experiments under the optimized chromatographic conditions. This approach allowed the validation and subsequent application of the method to quantify the target compounds in the selected THI. The LC–MS/MS method was validated in accordance with ICH guidelines [43], and the validation results are summarized in Table 3. Method validation and subsequent application were carried out using LyN-d_7_ as the IS, which was included in all analyses to correct for potential variations in extraction efficiency, sample preparation and instrumental response. This strategy minimizes matrix effects and improves the accuracy and precision of the quantification.

The selectivity of the method was confirmed by the absence of interfering peaks in blank extracts at the retention times of the analytes and LyN-d_7_, demonstrating that the optimized chromatographic conditions and MRM transitions provided selectivity despite the chemical complexity of THI. The relationships between the quantifier and qualifier transitions were also within ±20% of the expected values, supporting reliable analyte identification according to widely accepted MS/MS confirmation criteria. Linearity was evaluated using calibration curves starting at the concentration corresponding to the LOQ of each compound (Table 3). Correlation coefficients (R^2^) greater than 0.99 were obtained for Mc, Ht, HtN, RtN, Lc, and LcN, while values above 0.98 were obtained for Rt, Sc and ScN. The calibration curve for McN showed a lower coefficient of determination (R^2^ = 0.9666). However, satisfactory accuracy and precision were obtained for this compound, supporting its inclusion in the analytical method. Intra-day precision ranged between 1.43% and 8.17%, while inter-day precision ranged from 5.72 to 18.40%. Accuracy values varied from 83.5% to 111% for most compounds, while HtN and Sc showed values of 70.6 and 137% respectively.

Although these values fall outside common accepted accuracy criteria, similar values have been reported in other published LC-MS/MS methods for PAs in THI, where an accuracy below 80% or above 120% has been reported due to differences in matrix composition, extraction efficiency, and matrix-induced ionization effects [22,25,26,27,31,36]. Therefore, the accuracy values obtained in the present study are within the range of variability previously reported for these complex food matrices. Despite these limitations, the method demonstrated the capacity for simultaneous determination of the selected compounds.

The ME results showed that the matrix influenced the analytical response of the compounds under study. Seven of the ten analytes exhibited negative ME values, indicating varying degrees of signal suppression, whereas the remaining three showed signal enhancement. The most pronounced signal suppression was observed for Mc, Rt, and RtN, while McN, Ht, Sc, and ScN exhibited a moderate suppression. In contrast, HtN showed a slight increase in signal intensity, whereas Lc and LcN exhibited moderate and strong signal enhancement, respectively. The ME values were comparable to those reported for other analytical methods [25,50]. Although matrix effects were observed, the use of matrix-matched calibration curves accounted for these effects during analyte quantification, thereby improving the reliability of the analytical results.

The intra-day and inter-day precision values indicate that the method exhibits good repeatability and intermediate precision. Overall, the combined accuracy and precision results prove the suitability of the method for quantitative screening of the selected PAs and PANOs within the validated concentration ranges. The LOQ varied from 1.25 to 20.0 ng/g and the LOD ranged between 0.41 ng/g and 6.60 ng/g. These LODs and LOQ values were consistent with those reported in the literature [23,24,51]. Schulz et al. (2015) developed a method combining extraction with sulfuric acid-acidified water, followed by SPE using DSC-C18 SPE cartridges and LC-MS/MS analysis, achieving a LOQ of 10.0 ng/g [23]. Kwon et al. (2021) performed an extraction with MeOH and sulfuric acid followed by SPE clean-up using MCX cartridges and analysis by LC-MS/MS, with LODs ranging from 0.10 to 3.00 ng/g and LOQs from 0.30 to 9.00 ng/g [50], confirming that the present method provides comparable sensitivity while supporting broader matrix applicability.

### 3.4. Method Application

The method was applied to the quantification of target PAs and their PANOs in THI samples commercially available in the Portuguese and four other European countries market. Table 4 summarizes the detection frequency and concentration of the target compounds.

Mc was not detected in any sample, whereas McN was detected in one sample at a level below the LOQ. Ht and HtN were detected in six and seven samples, respectively. However, only two samples contained quantifiable levels of HtN, one of which contained 32.4 ng/g, representing one of the highest concentrations of the selected PAs measured. Lc was quantified in a single sample at 1.26 ng/g. Among compounds occurring in multiple samples, ScN was the most frequent (11 samples), with concentrations ranging from <LOQ to 13.9 ng/g. RtN was detected in eight samples, with concentrations between <LOQ and 31.2 ng/g. Sc was detected in five samples and showed the highest individual concentration observed in this study (53.2 ng/g).

The maximum concentration found in the present study was lower than those reported in other studies, which range from a few units to hundreds/thousands of ng/g depending on the plant and batch. Kwon et al. (2021) quantified several PAs in THI acquired from the South Korean market and detected concentrations of Sc ranging from ~20.0 to 300 ng/g [50]. Among the most frequently detected compounds, *N*-oxidized forms were the most prevalent, specifically ScN, RtN and HtN (*n* = 11, *n* = 8 and *n* = 7, respectively). This pattern is consistent with previous studies on THI in Europe, where *N*-oxide forms typically predominate, and Sc/Rt-type congeners are the most commonly detected. Other studies have also reported RtN and ScN as the predominant contaminants in THI, generally attributed to cross-contamination by *Senecio* spp. during harvesting [31,52]. Gumus (2023) reported that herbal infusions showed the highest total PA concentrations when compared to teas [53]. The PA profile was mainly characterized by senecionine- and retrorsine-type congeners, with ScN, RtN and HtN among the most frequently detected compounds. *N*-oxide forms predominated, a pattern consistent with findings from other European surveys and with our results [53].

Regarding the type of THI, as shown in the detailed analysis presented in Table 5, considerable variability was observed between product types. Eighteen of the 53 samples analyzed were positive for selected PAs and PANOs, of which 11 had total target PAs/PANOs values above the LOQs. Among these samples, herbal infusions had the highest number of positive selected PAs and PANOs detected. Concentrations ranged from values below the LOQs to 68.8 ng/g. Among the different types of herbal infusions, chamomile stood out due to its high detection frequency. Approximately 33% of samples with positive selected PAs/PANOs were chamomile, with concentrations between not detected to 68.8 ng/g, including two samples above 20.0 ng/g (PT013 and PT019). Lemon verbena also showed notable levels, with concentrations of 58.5 ng/g (PT008) and 34.1 ng/g (PT021). In contrast, traditional teas (green, black, and white) generally presented lower levels, ranging from 1.65 to 42.1 ng/g, with the highest concentration of target PAs/PANOs found in a black tea purchased on the Portuguese market (sample PT026).

These results align with general trends reported in the literature but reflect lower overall contamination levels. For instance, a previous study that analyzed 17 PAs in 282 THI samples from the German market using LC-MS/MS found the highest average concentration in rooibos tea (1856.40 µg/kg) and chamomile infusion (439.70 µg/kg), while black and green teas contained a mean value of 255.90 µg/kg and 109.00 µg/kg, respectively [46]. Griffin et al. (2014) similarly reported that infusions composed of mixed plants (e.g., chamomile with honey or spearmint) exhibited the highest PA concentrations (10.0–1438 µg/kg), whereas traditional teas (black, green, and yellow) showed lower levels (<LOQ to 19.0 µg/kg) [54]. The variability observed across studies likely reflects differences in geographical origin, botanical composition, harvesting season, and agricultural or manufacturing practices.

Although differences were observed in the detection frequency and concentration of selected PAs and PANOs among countries (Table 5), these results should be interpreted with caution. They may reflect variations in botanical composition, raw material origin, or manufacturing practices, as well as the limited and unbalanced number of samples analyzed per country. Moreover, the country of purchase does not necessarily correspond to the geographic origin of the raw material, since plant material used in THI may originate from different regions. The comparisons presented here should therefore be regarded as a preliminary reference rather than a representative cross-country assessment. This dataset is also not adequate for assessing dietary exposure specific to the Portuguese population, given the variability in product availability and consumption patterns across markets. A dietary exposure assessment was therefore not performed at this stage. The interpretation must also consider the scope of the target panel of analytes and the acidified extraction conditions. The concentrations described in Table 5 therefore represent the concentrations of the selected PAs/PANOs under the applied analytical conditions, not the total sum of PAs nor their concentrations in beverages prepared under typical household conditions. Future studies on dietary exposure to PAs should adopt a sample design aligned with the population under assessment and consider co-exposure to other tea and herbal infusion contaminants, which may modulate the overall risk associated with THI consumption. The preliminary occurrence data reported here will contribute to ongoing work aimed at assessing dietary exposure to target PAs and PANOs in the Portuguese population which will require a broader and population-representative sampling design.

## 4. Conclusions

This study developed and validated a sensitive and selective LC-MS/MS method for the determination of target PAs and their PANOs. The method was applied to the analysis of 53 THI samples marketed in different European countries as a pilot monitoring study. The method showed adequate sensitivity, selectivity, and performance according to international validation guidelines, enabling detection and screening of the target compounds at trace levels.

Of the 53 analyzed THI samples, 18 contained detectable levels of selected PAs/PANOs, predominantly in their *N*-oxide forms, with higher detection frequencies in herbal infusions, particularly chamomile and lemon verbena. Of these 18 positive samples, only 11 contained quantifiable levels, while the remaining 7 showed detectable concentrations but below the LOQ. The data obtained provide preliminary evidence of target PAs/PANOs occurrence in the analyzed THI samples, but further studies with larger sample sets are needed to better assess PA contamination in samples from European countries.

Although the concentrations were generally low, the detection of selected PAs and their PANOs in products regularly consumed by the population highlights the importance of further investigating their occurrence and potential contribution to dietary exposure, given the known hepatotoxic and genotoxic potential of these compounds.

These findings represent the data on the occurrence of target PAs and PANOs in some THI available in five European countries, primarily Portugal, where available data on occurrence remain limited and additional evidence is needed to support a more robust characterization.

## Figures and Tables

**Table 1:** Retention time, precursor ion (*m*/*z*) and MRM conditions for the selected PAs and PANOs (dwell time = 100 ms).

Analyte ^1^	Retention Time (min)	Precursor Ion (*m*/*z*)	MRM1		MRM2	
			Product Ion (*m*/*z*)	Collision Energy (eV)	Product Ion (*m*/*z*)	Collision Energy (eV)
LyN-d_7_	15.02	323.10	172.00	−30.0	-	-
Ht	17.52	314.30	138.10	−21.0	156.10	−27.0
HtN	18.99	330.30	172.00	−28.0	111.15	−43.0
Lc	25.46	412.40	120.10	−30.0	336.10	−21.0
LcN	27.95	428.40	254.10	−30.0	94.05	−50.0
Mc	8.43	326.30	120.05	−38.0	121.15	−30.0
McN	12.76	342.30	137.05	−30.0	119.00	−35.0
Rt	16.37	352.30	120.05	−32.0	138.15	−32.0
RtN	17.40	368.30	94.10	−48.0	120.10	−35.0
Sc	20.34	336.30	120.10	−32.0	94.15	−34.0
ScN	21.77	352.30	94.00	−46.0	120.10	−40.0

^1^ Ht: Heliotrine; HtN: Heliotrine *N*-oxide; Lc: Lasiocarpine; LcN: Lasiocarpine *N*-oxide; LyN-d7: (+)—Lycopsamine-*N*-oxide-d_7_; Mc: Monocrotaline; McN: Monocrotaline *N*-oxide; Rt: Retrorsine; RtN: Retrorsine *N*-oxide; Sc: Senecionine; ScN: Senecionine *N*-oxide.

**Table 2:** Summary of the different QuEChERS extraction protocols evaluated ^1^.

	QuEChERS Protocol 1	QuEChERS Protocol 2	QuEChERS Protocol 3
Sample (mL)	10 mL	10 mL	1 mL
Extraction solvent and volume (mL)	ACN (10 mL)	ACN/ammonium carbonate (200 mg L^−1^);	ACN (1 mL)
		84:16 (*v*/*v*, pH 8.5) solution (10 mL)	
Stirring time (min)	3 min	15 min	30 min
Centrifugation conditions (time, g)	10 min; 4000 g	20 min; 7412 g	-
Salt composition (g)	MgSO_4_ (4 g)	-	MgSO_4_ (0.4 g)
	NaCl (1 g)		NaCl (0.1 g)
	Trisodium citrate dihydrate (1 g)		Trisodium citrate dihydrate (0.1 g)
	Disodium citrate sesquihydrate (0.5 g)		Disodium citrate sesquihydrate (0.05 g)
Clean-up sorbent (mg)	-	PSA (50 mg)	MgSO_4_ (150 mg)
			PSA (25 mg)

^1^ ACN: Acetonitrile; MgSO_4_: anhydrous magnesium sulphate; NaCl: Sodium chloride; PSA: primary secondary amine.

**Table 3:** Linearity parameters, LOQ and LOD, intra-day and inter-day precision, accuracy and matrix effect for the target PAs and PANOs.

Analyte ^1^	Range (ng/g)	Linear Regression	R^2^	LOD (ng/g)	LOQ (ng/g)	Intra-Day Precision (% RSD)	Inter-Day Precision (% RSD)	Accuracy (%)	Matrix Effect (%)
Ht	5.00–80.0	y = 3.1661x + 0.0422	0.9973	1.65	5.00	[2.87–5.14]	6.52	[97.6–98.7]	−25.68
HtN	5.00–80.0	y = 2.246x + 0.0825	0.9905	1.65	5.00	[1.43–4.54]	7.30	[70.6–109]	8.71
Lc	1.25–80.0	y = 3.5788x − 0.005	0.9977	0.41	1.25	[5.47–7.89]	16.70	[88.0–111]	28.92
LcN	1.25–80.0	y = 0.9304x + 0.0006	0.9972	0.41	1.25	[4.61–5.81]	18.40	[83.5–107]	67.91
Mc	5.00–80.0	y = 0.4426x + 0.0127	0.9967	1.65	5.00	[5.26–8.06]	11.16	[92.8–110]	−67.69
McN	10.0–80.0	y = 0.5336x + 0.0426	0.9666	3.30	10.0	[3.23–5.46]	7.79	[85.3–105]	−51.33
Rt	5.00–80.0	y = 0.2463x − 0.0048	0.9886	1.65	5.00	[3.29–7.67]	9.86	[83.7–97.1]	−60.36
RtN	5.00–80.0	y = 0.3605x + 0.0069	0.9951	1.65	5.00	[2.49–4.14]	7.39	[85.8–92.3]	−63.85
Sc	20.0–80.0	y = 0.1397x − 0.003	0.9871	6.60	20.0	[6.00–8.17]	8.40	[92.1–137]	−37.77
ScN	5.00–80.0	y = 0.8277x + 0.0072	0.9827	1.65	5.00	[3.55–4.90]	5.72	[87.1–87.7]	−26.70

^1^ Ht: Heliotrine; HtN: Heliotrine *N*-oxide; Lc: Lasiocarpine; LcN: Lasiocarpine *N*-oxide; Mc: Monocrotaline; McN: Monocrotaline *N*-oxide; Rt: Retrorsine; RtN: Retrorsine *N*-oxide; Sc: Senecionine; ScN: Senecionine *N*-oxide.

**Table 4:** Detection frequency and concentration levels of targeted PAs and PANOs.

Target PA ^1^	Frequency of Positive Samples	Minimum Concentration [ng/g]	Maximum Concentration [ng/g]
Ht	6	<LOQ	<LOQ
HtN	7	<LOQ	32.4
Lc	1	-	1.26
LcN	1	-	1.55
Mc	0	-	-
McN	1	-	<LOQ
Rt	2	14.0	17.4
RtN	8	<LOQ	31.2
Sc	5	<LOQ	53.2
ScN	11	<LOQ	13.9

^1^ Ht: Heliotrine; HtN: Heliotrine *N*-oxide; Lc: Lasiocarpine; LcN: Lasiocarpine *N*-oxide; Mc: Monocrotaline; McN: Monocrotaline *N*-oxide; Rt: Retrorsine; RtN: Retrorsine *N*-oxide; Sc: Senecionine; ScN: Senecionine *N*-oxide.

**Table 5:** Concentration of individual PAs and their PANOs ^1^ determined in the positive THI samples (expressed in ng/g dry weight).

Sample	THI Type	Ht	HtN	Lc	LcN	Mc	McN	Rt	RtN	Sc	ScN	Total Target PAs/PANOs Content (ng/g) *
PT007	Chamomile	-	-	-	-	-	-	-	-	-	6.91	6.91
PT010	Chamomile	-	-	-	-	-	-	-	6.42	<LOQ	<LOQ	14.7
PT013	Chamomile	-	-	-	-	-	-	-	<LOQ	53.2	13.9	68.8
PT019	Chamomile	-	-	-	-	-	-	-	13.4	<LOQ	5.38	25.4
SP002	Chamomile	-	-	-	-	-	-	-	-	-	<LOQ	1.65
IT003	Chamomile	-	-	-	-	-	-	-	-	-	<LOQ	1.65
AU001	Pepermint	-	-	-	-	-	-	-	<LOQ	<LOQ	<LOQ	9.90
PT002	Lemon Verbena	<LOQ	<LOQ	-	-	-	-	-	-	-	-	3.30
PT008	Lemon Verbena	<LOQ	<LOQ	-	-	-	-	17.4	31.2	<LOQ	-	58.5
PT016	Lemon Verbena	<LOQ	6.17	-	-	-	-	-	-	-	-	7.82
PT021	Lemon Verbena	<LOQ	32.4	-	-	-	-	-	-	-	-	34.1
PT023	Lemon Balm	<LOQ	<LOQ	1.26	1.55	-	-	-	-	-	<LOQ	7.76
PT001	Green tea	-	<LOQ	-	-	-	-	-	<LOQ	-	-	3.30
PT006	Green tea	<LOQ	<LOQ	-	-	-	<LOQ	-	-	-	-	6.60
IT001	Green tea	-	-	-	-	-	-	-	6.68	-	-	6.68
PT026	Black tea	-	-	-	-	-	-	14.0	26.4	-	<LOQ	42.1
FR001	Black tea	-	-	-	-	-	-	-	-	-	5.95	5.95
PT014	White tea	-	-	-	-	-	-	-	-	-	<LOQ	1.65

Ht: Heliotrine; HtN: Heliotrine *N*-oxide; Lc: Lasiocarpine; LcN: Lasiocarpine *N*-oxide; Mc: Monocrotaline; McN: Monocrotaline *N*-oxide; Rt: Retrorsine; RtN: Retrorsine *N*-oxide; Sc: Senecionine; ScN: Senecionine *N*-oxide. * Total target PA/PANO content was estimated considering the sum of all targeted compounds, where the LOD value was considered for those below LOQ.

## Data Availability

The original contributions presented in this study are included in the article/Supplementary Materials. Further inquiries can be directed to the corresponding authors.

## Supplementary Materials

The following supporting information can be downloaded at: https://www.mdpi.com/article/10.3390/foods15203598/s1, Table S1: Overview of the most recent analytical methods for determination of PAs/PANOs in flora and food products [25,26,27,28,29,30,31,32,33,34,35,36]; Table S2: Description of the 53 tea and herbal infusion (THI) samples analyzed in the present study, including label information and country of acquisition; Table S3: Chromatographic conditions used during HPLC-UV/Vis method optimization; Figure S1: Chromatographic characterization of selected PAs and PANOs under different conditions.

## Abbreviations

The following abbreviations are used in this manuscript:

A_A_	Analyte peak area
ACN	Acetonitrile
A_IS_	Internal standard peak area
BfR	German Federal Institute for Risk Assessment
BMDL_10_	Benchmark Dose Lower Confidence Limit for a 10% response
CE	Collision Energy
CID	Collision-Induced Dissociation
EFSA	European Food Safety Authority
ESI	Electrospray Ionization
EtOH	Ethanol
FA	Formic Acid
Ht	Heliotrine
HtN	Heliotrine *N*-oxide
IARC	International Agency for Research on Cancer
ICH	International Council for Harmonisation
IS	Internal Standard
LC-MS/MS	Liquid Chromatography–Tandem Mass Spectrometry
LC-UV/Vis	Liquid Chromatography–Ultraviolet/Visible
Lc	Lasiocarpine
LcN	Lasiocarpine *N*-oxide
LOD	Limit of Detection
LOQ	Limit of Quantification
LyN-d_7_	Lycopsamine-*N*-oxide-d_7_
Mc	Monocrotaline
McN	Monocrotaline *N*-oxide
MCX	Mixed-Mode Cation Exchange
ME	Matrix Effect
MeOH	Methanol
MgSO_4_	Magnesium sulphate
MRM	Multiple Reaction Monitoring
MRM1	Primary Multiple Reaction Monitoring transition (quantifier ion)
MRM2	Secondary Multiple Reaction Monitoring transition (qualifier ion)
NaCl	Sodium chloride
PANOs	Pyrrolizidine Alkaloid *N*-oxides
PAs	Pyrrolizidine Alkaloids
PFP	Pentafluorophenyl
PSA	Primary Secondary Amine
QC	Quality Control
QuEChERS	Quick, Easy, Cheap, Effective, Rugged and Safe
rpm	revolutions per minute
RSD	Relative Standard Deviation
Rt	Retrorsine
RtN	Retrorsine *N*-oxide
Sc	Senecionine
ScN	Senecionine *N*-oxide
SPE	Solid-Phase Extraction
THI	Teas and Herbal Infusions
UHPLC-MS/MS	Ultra-High Performance Liquid Chromatography–Tandem Mass Spectrometry
UPW	Ultrapure Water
