# Supplementary material for: Determination of Pyrrolizidine Alkaloids in Tea and Herbal Infusions by LC-MS/MS
Source: Foods. 2026 Oct 10;15(20):3598. doi: 10.3390/foods15203598 (PMC13704718; doi:10.3390/foods15203598)
Supplement: Supplementary file 1 [file foods-15-03598-s001.zip › foods-4532994-supplementary.pdf]

Supplementary Material for:

Determination of Pyrrolizidine Alkaloids in Tea and Herbal infusions by LC-MS/MS

Ana Catarina Sousa [1,2], Daniela Pereira [3], Ana R.L. Ribeiro [3], Virgínia M.F. Gonçalves [1,4,5], Inês Pádua [1,4], Cláudia Ribeiro [1,4,*], Sandra Leal [1,2,*]

[1] Associate Laboratory i4HB-Institute for Health and Bioeconomy, CESPU University, 4585-116 Gandra, Portugal

[2] UCIBIO-Research Unit on Applied Molecular Biosciences, Toxicologic Pathology Research Laboratory, CESPU University, 4585-116 Gandra, Portugal

[3] LSRE-LCM, ALiCE, Faculty of Engineering, University of Porto, 4200-465 Porto, Portugal

[4] UCIBIO-Research Unit on Applied Molecular Biosciences, Translational Toxicology Research Laboratory, CESPU University, 4585-116 Gandra, Portugal

[5] UNIPRO–Oral Pathology and Rehabilitation Research Unit, CESPU University, 4585-116 Gandra, Portugal

*Corresponding Authors:

E-mail: claudia.ribeiro@iucs.cespu.pt (C.R.), sandra.leal@iucs.cespu.pt (S.L.)

Table of Contents

Table S1: Overview of the most recent analytical methods for determination of PAs/PANOs in flora and food products

Table S2: Description of the 53 tea and herbal infusion (THI) samples analyzed in the present study, including label information and country of acquisition.

Table S3: Chromatographic conditions used during HPLC-UV/Vis method optimization

Figure S1: Chromatographic characterization of selected PAs and PANOs under different conditions

References

Table S1: Overview of the most recent analytical methods for determination of PAs/PANOs in flora and food products

Author, year [ref])	Sample	PAs and PANOs	Extraction solvent	Sample preparation technique	Analytical method	Recovery (R) (%) / Extraction efficiencies (EE) (%)	LOD /LOQ/LOI (ng/g)
**Carbonell-Rozas et al. 2025 [26]**	Teas, herbal infusions, aromatic herbs and honey	Intermedine, Rinderine, Indicine, Lycopsamine, Europine, Spartiodine, Retrorsine, Heliotrine, Seneciphylline, Integerrimine, Senecionine, Echimidine, Lasiocarpine, Usaramine, Heliosupine, Senecivernine and their N-oxide forms; Senkirkine	MeOH	SPE (Oasis MCX cartridge)	LC-MS/MS	R: 62.0 - 103.0	LOD: 0.02–1.20
**Jiao et al. 2024 [27]**	Weeds, soil samples, and fresh tea leaves	Heliotrine, Retrorsine, Senecionine, Jacobine, Intermedine, Seneciphylline, Europine and their N-oxide forms, Senkirkine	0.1 M H_2SO_4	QuEChERS based	UPLC-MS/MS	R: 72.04 - 114.17	LOQ: 20.0
**Sallam et al. 2026 [28]**	Medicinal plants and herbal teas	Echimidine, Erucifoline, Europine, Heliotrine, Intermedine, Jacobine, Lasiocarpine, Lycopsamine, Monocrotaline, Retrorsine, Senecionine, Seneciphylline, Senecivernine and their N-oxides forms; Senkirkine and Trichodesmine	H_2O and ACN	QuEChERS	LC-MS/MS	R: 70.00 - 120.0	LOQ: 10.0

**Abdallah et al. 2026 [29]**	Tea and herbal infusion	Heliotrine, Lycopsamine, Senecionine N-oxide, Jacobine N-oxide, Intermedine N-oxide and Retorsine N-oxide	0.05 M H_2SO_4	SPE (DSC-C18 SPE)	UPLC-MS/MS	n.d.	LOD: 0.18–0.75; LOQ: 0.48 - 2.27
**Lin et al. 2025 [30]**	Teas, honey, milk, spices, and Chinese medicines	Intermedine, Retrorsine, Jacobine, Lycopsamine, Riddelliine, Seneciphylline, Echimidine, Monocrotaline, Senecionine, Lasiocarpine, Heliotrine and their N-oxide forms; Senkirkine and Petasitenine	% solution of formic acid	SPE (PCX cartridge)	UHPLC-MS/MS	R: 64.5 - 112.2	LOD: 0.015 - 0.75; LOQ: 0.05 - 2.5
**León et al. 2022 [31]**	Teas and herbal infusion	Echimidine, Erucifoline, Europine, Heliotrine, Intermedine, Lycopsamine, Jacobine, Lasiocarpine, Monocrotaline, Retrorsine, Senecionine, Seneciphylline and their N-oxide forms; Senkirkine and Trichodesmine	H_2O and ACN	QuEChERS	LC-Q-Orbitrap HRMS	R: 87.0 - 106.0	LOQ: 5.00
**Peloso et al. 2023 [32]**	Fresh leaves, dried herbs, teas and herbal infusion, honey and pollen	Echimidine, Echinatine, Europine, Heliosupine, Heliotrine, Indicine, Integerrimine, Intermedine, Lasiocarpine, Lycopsamine, Retrorsine, Rinderine, Senecionine, Seneciphylline, Senecivernine, Spartioidine, Usaramine	0.1 M H_2SO_4	SPE (Oasis MCX cartridge)	LC-MS/MS	R: 70.2 - 103.5	LOQ: 1.00 - 5.00

		and their N-oxide forms; Senkirkine					
**Jansons et al. 2022 [33]**	Honey, tea, herbal tinctures, and milk	Echimidine, Echinatine, Europine hydrochloride, Heliosupine, Heliotrine, Indicine hydrochloride, Integerrimine, Intermedine, Lasiocarpine, Lycopsamine, Retrorsine, Senecionine, Seneciphylline, Senecivernine and their N-oxide forms; Senkirkine and Usaramine	0.2% formic acid	QuEChERS and SPE (SCX cartridge)	Nano-LC-MS	n.d.	LOQ: 0.05 – 10.0
**Rizzo et al. 2023 [34]**	Food supplements, herbal infusions, honey and teas	Echimidine, Erucifoline, Europine, Heliotrine, Intermedine, Jacobine, Lasiocarpine, Lycopsamine, Monocrotaline, Retrorsine, Senecionine, Seneciphylline, Senecivernine and their N-oxides forms; Senkirkine and Trichodesmine	0.05 M H_2SO_4	SALLE	UHPLC-HRMS/MS	EE: 63.0 - 107.9	LOQ: 0.10 - 12.0

**Rizzo et al. 2023 [35]**	Plant materials, honey, pollen and herbal infusion	Echimidine, Erucifoline, Europine, Heliotrine, Indicine, Intermedine, Jacobine, Lasiocarpine, Lycopsamine, Monocrotaline, Retrorsine, Senecionine, Seneciphylline, Senecivernine and their N-oxide forms; Senkirkine and Trichodesmine	0.05 M H_2SO_4	SALLE	UHPLC-HRMS	EF: 68.8 - 109.1	LOI: 0.60 - 20.0
**Luo et al. 2022 [36]**	Tea	Lasiocarpine, Heliotrine, Monocrotaline, Erucifoline, Senecionine, Europine, Seneciphylline, Echimidine, Retrorsine and Lycopsamine	MeOH and 0.05 mM H_2SO_4	n.d.	UPLC-Q-TOF-MS	R: 90.7 - 107.6	LOD: 0.08 - 0.54 ng/mL; LOQ: 0.26 - 1.77 ng/mL
**Chung et al. 2018 [37]**	Food	Echimidine, Erucifoline, Europine, Heliotrine, Intermedine, Jacobine, Lasiocarpine, Lycopsamine, Monocrotaline, Retrorsine, Senecionine, Seneciphylline, Senecivernine and their N-oxides forms; Senkirkine and Trichodesmine	0.05 M H_2SO_4	SPE (C18) cartridges	UPLC-MS/MS	R: 80.0 - 155.3	LOQ: 0.01 - 0.09

ACN- Acetonitrile; **EE**- Extraction efficiencies **LOD**- Limit of detection; **LOI**- Limit of identification; **LOQ**- Limit of quantification; **MCX**- Mixed-mode cation exchange; **n.d.**: not described; **PCX**- polymeric cation exchange; **QuEChERS**- Quick Easy Cheap Effective Rugged Safe; **SALLE-** Salting-out assisted liquid–liquid extraction; **SCX**- Strong cation exchanger; **SPE**- Solid phase extraction; **MeOH-** Methanol; **H_2O-** Water; **H_2SO_4-** Sulfuric Acid

Table S2: Description of the 53 tea and herbal infusion (THI) samples analyzed in the present study, including label information and country of acquisition.

ID	Type of THI	Ingredients (% by weight) (as a header label)	Place of Purchase (city, country)	Expiry date	Quantity per bag (g)	Flavoring	Country of origin (as a header label)
PT001	Green tea	100% Green tea	Porto, Portugal	Sep-25	1.3g	no	NS
PT002	Lemon verbena	100% Lemon verbena	Porto, Portugal	Dec-25	1.3g	no	NS
PT003	Chamomile	100% Chamomile	Porto, Portugal	Jan-26	0.7g	no	NS
PT004	Chamomile	100% Chamomile	Porto, Portugal	Apr-27	1.2g	no	NS
PT005	Green tea	100% Green tea	Porto, Portugal	Mar-27	1.5g	no	NS
PT006	Green tea	100% Green tea	Porto, Portugal	Jan-27	1.3g	no	NS
PT007	Chamomile	100% Chamomile	Porto, Portugal	Sep-26	1.2g	no	NS
PT008	Lemon verbena	100% Lemon verbena	Porto, Portugal	Oct-26	1.5g	no	NS
PT009	Green tea	100% Green tea	Porto, Portugal	May-25	2g	no	NS
PT010	Chamomile	100% Chamomile	Porto, Portugal	Mar-27	1.2g	no	NS
PT011	Green tea	100% Green tea	Porto, Portugal	Mar-27	1.8g	no	NS
PT012	Green tea	100% Green tea	Porto, Portugal	Feb-27	1.5g	no	NS
PT013	Chamomile	100% Chamomile	Porto, Portugal	Feb-27	1.35g	no	NS
PT014	White tea	95% White tea; 5% vanilla flavouring	Porto, Portugal	Feb-27	1.5g	yes	NS
PT015	Lemon balm	100% Lemon balm	Porto, Portugal	Mar-27	1.5g	no	NS
PT016	Lemon verbena	100% Lemon verbena	Porto, Portugal	Dec-26	1.3g	no	NS
PT017	Green tea	NS	Porto, Portugal	Mar-27	1.5g	no	NS
PT018	Green tea	NS	Porto, Portugal	Mar-27	1.5g	no	NS
PT019	Chamomile	NS	Porto, Portugal	Apr-27	1.2g	no	NS
PT020	Green tea	100% Green tea	Porto, Portugal	Apr-26	1.5g	no	Poland

PT021	Lemon verbena	100% Lemon verbena	Porto, Portugal	Jul-25	1.3g	no	Spain
PT022	Chamomile	100% Chamomile	Porto, Portugal	Mar-26	1.35g	no	Albania
PT023	Lemon balm	100% Lemon balm	Porto, Portugal	Oct-25	1.5g	no	Spain
PT024	Black tea	100% Black tea	Porto, Portugal	Nov-27	1.5g	no	EU
PT025	Black tea	100% Black tea	Porto, Portugal	Dez-28	1.5g	no	NS
PT026	Black tea	100% Black tea	Porto, Portugal	Feb-28	1.8g	no	NS
FR001	Herbal mix	Black tea; 3% bergamot flavoring	Paris, France	Feb-26	2g	yes	China
FR002	Herbal mix	58.5% Black tea; 24% hibiscus. raspberry fruit; 16% stevia	Paris, France	Sep-26	2g	no	France
FR003	Herbal mix	51% Green tea; 42% Mint; natural flavoring	Paris, France	Jan-27	1.8g	yes	France
FR004	Herbal mix	23% black and green tea; 51% lemon fruit; 26% white hibiscus	Paris, France	Feb-27	2.04g	no	UE
FR005	Herbal mix	95% Green tea; 5% ginger natural flavoring	Paris, France	Mar-26	NS	yes	NS
FR006	Herbal mix	Black tea; 20% flavoring; 1.5% peach;1.4% mango	Paris, France	NS	NS	yes	NS
FR007	Herbal mix	44% Green tea; 2% Mate tea; Rooibos tea	Paris, France	Mar-27	1.5g	no	NS
FR008	Herbal mix	Green tea; vanilla and red fruit flavoring	Paris, France	Oct-27	2.7g	yes	NS
FR009	Herbal mix	White tea; 7%. lemon flavoring; 2% lemon fruit	Paris, France	Aug-25	1.7g	yes	NS
SP001	Chamomile	100% Chamomile (flower)	Madrid, Spain	Apr-26	1.1g	no	Amsterdam
SP002	Chamomile	100% Chamomile (flower)	Madrid, Spain	NS	1.2g	no	Alicante
SP003	Chamomile	100% Chamomile (flower)	Madrid, Spain	Apr-27	1.1g	no	Alicante
SP004	Green tea	100% Green tea	Barcelona, Spain	Apr-26	1.5g	no	Amsterdam
SP005	Green tea	100% Green tea	Madrid, Spain	Jan-27	1.5g	no	Alicante
SP006	Green tea	100% Green tea	Madrid, Spain	Apr-27	1.5g	no	Alicante
SP007	Mint	99% Metha piperita. 1% Mentha pulegium	Barcelona, Spain	NS	1.4g	no	Amsterdam
SP008	Mint	100% Mint	Madrid, Spain	Mar-27	1.5g	no	Alicante

SP009	Mint	88% Metha piperita; 12% Mentha pulegium	Madrid, Spain	Mar-27	1.5g	no	Alicante
SP010	Black tea	100% Black tea	Barcelona, Spain	Sep-25	1.75g	no	Amsterdam
SP011	Maca	20% Rooibos and cinnamon; 12% maca; 10% aniseed and ginger; natural flavorings	Barcelona, Spain	Jan-26	1.7g	yes	Amsterdam
IT001	Green tea	100% Green tea	Vercelli, Italy	Mar-27	1.6g	no	NS
IT002	Black tea	100% Black tea	Vercelli, Italy	Apr-27	1.5g	no	NS
IT003	Chamomile	100% Chamomile	Vercelli, Italy	NS	1.67g	no	NS
AU001	Pepermint	100% Pepermint	Vienna, Austria	Jan-28	1.75g	no	NS
AU002	Chamomile	100% Chamomile	Vienna, Austria	Feb-28	1.4g	no	NS
AU003	Pepermint	100% Pepermint	Vienna, Austria	Jan-27	1.5g	no	NS
AU004	Green tea	100% Green tea	Vienna, Austria	Dec-27	1.5g	no	NS

EU: European Union; NS: Not stated; mix: mixtures

Table S3: Chromatographic conditions used during HPLC-UV/Vis method optimization.

	Eluent A	%	Eluent B	%	Eluent C	%	Type of elution	Flow rate (mL/min)	T column oven (°C)	Wavelength
1	10 mM ammonium formate with 0.1% formic acid; pH = 4	70	ACN	30	–	–	Isocratic elution	0.7	25	223
2	10 mM ammonium formate with 0.1% formic acid; pH = 4	20	ACN	80	–	–	Isocratic elution	0.7	25	223
3	10 mM ammonium formate with 0.1% formic acid; pH = 4	30	EtOH	70	–	–	Isocratic elution	0.5	25	223
4	10 mM ammonium formate with 0.1% formic acid; pH = 4	40	EtOH	60	–	–	Isocratic elution	0.4	40	223
5	10 mM ammonium formate with 0.1% formic acid; pH = 4	50	EtOH	50	–	–	Isocratic elution	0.4	35	223
6	10 mM ammonium formate with 0.1% formic acid; pH = 4	45	EtOH	55	–	–	Isocratic elution	0.4	25	223
7	10 mM ammonium formate with 0.1% formic acid	40	EtOH	40	ACN	20	Isocratic elution	0.4	25	235
8	10 mM ammonium formate with 0.1% formic acid; pH = 4	40	EtOH	50	ACN	10	Isocratic elution	0.4	25	235
9	10 mM ammonium formate with 0.1% formic acid; pH = 4	40	EtOH	55	ACN	5	Isocratic elution	0.4	25	235

10	10 mM ammonium formate with 0.1% formic acid; pH = 4	40	EtOH	58	ACN	2	Isocratic elution	0.4	25	235
11	10 mM ammonium formate with 0.1% formic acid; pH=4	40	EtOH	58	ACN	2	Isocratic elution	0.35	25	235
12	10 mM ammonium formate with 0.1% formic acid; pH = 4	60	EtOH	40	–	–	Gradient elution	0.4	25	235
13	10 mM ammonium formate with 0.1% formic acid; pH = 4	65	EtOH	35	–	–	Gradient elution	0.4	25	235
14	10 mM ammonium formate with 0.1% formic acid; pH = 4	55	EtOH	45	–	–	Gradient elution	0.4	25	235
15	10 mM ammonium formate with 0.1% formic acid; pH = 4	70	EtOH	30	–	–	Gradient elution	0.4	25	235
16	10 mM ammonium formate with 0.1% formic acid; pH = 4	80	EtOH	20	–	–	Gradient elution	0.4	25	235
17	10 mM ammonium formate with 0.1% formic acid; pH = 4	85	EtOH	15	–	–	Gradient elution	0.4	25	235
18	10 mM ammonium formate with 0.1% formic acid; pH = 4	85	EtOH	15	–	–	Gradient elution	0.5	25	235
19	20 mM ammonium formate; pH = 6.6	85	EtOH	15	–	–	Gradient elution	0.4	25	235
20	20 mM ammonium formate with 0.1% formic acid; pH = 5	85	EtOH	15	–	–	Gradient elution	0.4	25	235

20	10 mM ammonium formate with 0.1% formic acid; pH = 4	85	EtOH	10	MeOH	5	Gradient elution	0.4	25	235
21	10 mM ammonium formate with 0.1% formic acid; pH = 4	85	EtOH	9	MeOH	6	Gradient elution	0.4	25	235

ACN: acetonitrile; EtOH: ethanol; MeOH: methanol. Under all the conditions described, 10 µL was used as the injection volume.

A

B

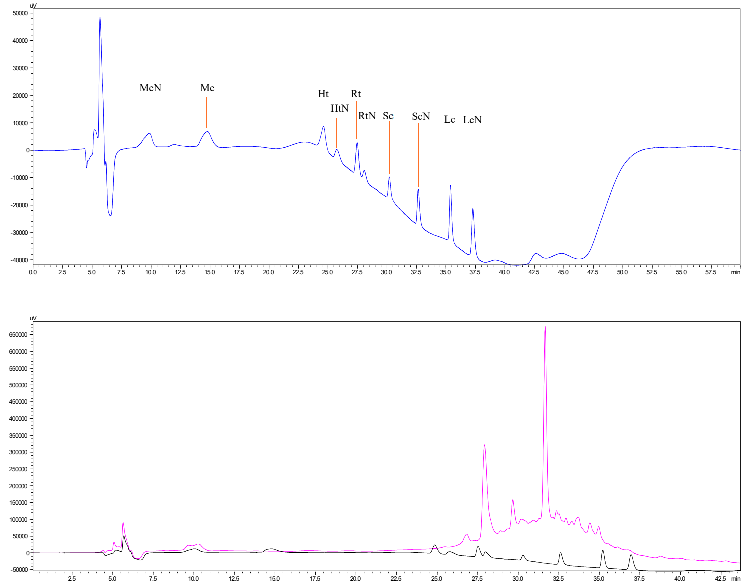

Figure S1: Chromatographic characterization of selected PAs and PANOs under different conditions. **(A)** Chromatogram of a standard mixture prepared in EtOH, containing 10 µg/mL of Lc, LcN, Rt, RtN, Sc, and ScN, and 100 µg/mL of Ht, HtN, Mc, and McN. The analysis was performed using a mobile phase composed of 10 mM ammonium formate in UPW with 0.1% formic acid: EtOH: MeOH in a ratio of 85:9:6 (*v/v/v*), at a flow rate of 0.4 mL/min. The wavelength was set at 235 nm. **(B)** Overlay chromatograms of a tea sample spiked with a mixture of standards (pink line) and a standard mixture prepared in EtOH, containing 10 µg/mL of Lc, LcN, Rt, RtN, Sc, and ScN, and 100 µg/mL of Ht, HtN, Mc, and McN (black line). The analysis was performed using a mobile phase composed of 10 mM ammonium formate in UPW with 0.1% formic acid: EtOH: MeOH (85:9:6, *v*/*v*/*v*), at a flow rate of 0.4 mL/min, and wavelength of 235 nm. Ht: Heliotrine; HtN: Heliotrine *N*-oxide; Lc: Lasiocarpine; LcN: Lasiocarpine *N*-oxide; Mc: Monocrotaline; McN: Monocrotaline *N*-oxide; Rt: Retrorsine; RtN: Retrorsine *N*-oxide; Sc: Senecionine; ScN: Senecionine *N*-oxide.

References

26. Carbonell-Rozas, L.; Dreolin, N.; Foddy, H.; Dall'Asta, C. Enhancing Pyrrolizidine Alkaloid Separation and Detection: LC-MS/MS Method Development and Integration of Ion Mobility Spectrometry into the LC-HRMS Workflow. *J. Chromatogr. A* **2025**, *1748*, 465863. https://doi.org/10.1016/J.CHROMA.2025.465863.

27. Jiao, W.; Zhu, L.; Shen, T.; Wang, L.; Li, Q.X.; Wang, C.; Wu, X.; Chen, H.; Hua, R. Simultaneous Determination of 15 Pyrrolizidine Alkaloids and Their N-Oxides in Weeds, Soil, Fresh Tea Leaves, and Tea: Exploring the Pollution Source of Pyrrolizidine Alkaloids in Tea. *Food Chem.* **2024**, *434*. https://doi.org/10.1016/J.FOODCHEM.2023.137305.

28. Sallam, M.; Shallan, M.A.; Mohamed, M.A.; El-Sawi, S.A.M.; Abdelwahed, M.H. Simultaneous Method Validation and Surveillance of Pyrrolizidine Alkaloids and Their N-Oxides in Some Medicinal Plants and Herbal Teas Using QuEChERS–LC-MS/MS. *Talanta* **2026**, *309*. https://doi.org/10.1016/j.talanta.2026.130022.

29. Abdallah, L.A.; Merhi, A.; Hayeck, N.; Shaib, H.; Abiad, M.G.; Obeid, O.; Iskandar, C.F. Pyrrolizidine Alkaloid Contamination in Loose Tea and Its Health Risk Assessment. *Food and Chemical Toxicology* **2026**, *207*, 115826. https://doi.org/10.1016/j.fct.2025.115826.

30. Lin, R.; Peng, J.; Zhu, Y.; Dong, S.; Jiang, X.; Shen, D.; Li, J.; Zhu, P.; Mao, J.; Wang, N.; et al. Quantitative Analysis of Pyrrolizidine Alkaloids in Food Matrices and Plant-Derived Samples Using UHPLC—MS/MS. *Foods* **2025**, *14*, 1147. https://doi.org/10.3390/foods14071147.

31. León, N.; Miralles, P.; Yusà, V.; Coscollà, C. A Green Analytical Method for the Simultaneous Determination of 30 Tropane and Pyrrolizidine Alkaloids and Their N-Oxides in Teas and Herbs for Infusions by LC-Q-Orbitrap HRMS. *J. Chromatogr. A* **2022**, *1666*. https://doi.org/10.1016/j.chroma.2022.462835.

32. Peloso, M.; Minkoumba Sonfack, G.; Paduano, S.; De Martino, M.; De Santis, B.; Caprai, E. Pyrrolizidine Alkaloids in Food on the Italian Market. Molecules **2023**, 28, 5346. https://doi.org/10.3390/molecules28145346.

33. Jansons, M.; Fedorenko, D.; Pavlenko, R.; Berzina, Z.; Bartkevics, V. Nanoflow Liquid Chromatography Mass Spectrometry Method for Quantitative Analysis and Target Ion Screening of Pyrrolizidine Alkaloids in Honey, Tea, Herbal Tinctures, and Milk. *J. Chromatogr. A* **2022**, *1676*, 463269, https://doi.org/10.1016/j.chroma.2022.463269.

34. Rizzo, S.; Celano, R.; Piccinelli, A.L.; Russo, M.; Rastrelli, L. Target Screening Method for the Quantitative Determination of 118 Pyrrolizidine Alkaloids in Food Supplements, Herbal Infusions, Honey and Teas by Liquid Chromatography Coupled to Quadrupole Orbitrap Mass Spectrometry. *Food Chem.* **2023**, *423*, 136306. https://doi.org/10.1016/j.foodchem.2023.136306.

35. Rizzo, S.; Celano, R.; Piccinelli, A.L.; Serio, S.; Russo, M.; Rastrelli, L. An Analytical Platform for the Screening and Identification of Pyrrolizidine Alkaloids in Food Matrices with High Risk of Contamination. *Food Chem.* **2023**, *406*, 135058. https://doi.org/10.1016/j.foodchem.2022.135058.

36. Luo, Z.; Chen, X.; Ma, Y.; Yang, F.; He, N.; Yu, L.; Zeng, A. Multi-Template Imprinted Solid-Phase Microextraction Coupled with UPLC-Q-TOF-MS for Simultaneous Monitoring of Ten Hepatotoxic Pyrrolizidine Alkaloids in Scented Tea. *Front. Chem.* **2022**, *10*. https://doi.org/10.3389/fchem.2022.1048467.

37. Chung, S.W.C.; Lam, C.H. Development of an Analytical Method for Analyzing Pyrrolizidine Alkaloids in Different Groups of Food by UPLC-MS/MS. *J. Agric. Food Chem.* **2018**, *66*, 3009–3018. https://doi.org/10.1021/ACS.JAFC.7B06118.
